# Maximizing multiple influences and fair seed allocation on multilayer social networks

**DOI:** 10.1371/journal.pone.0229201

**Published:** 2020-03-12

**Authors:** Yu Chen, Wei Wang, Jinping Feng, Ying Lu, Xinqi Gong

**Affiliations:** 1 School of Mathematics, Renmin University of China, Beijing, China; 2 School of Mathematics and Statistics, Minnan Normal University, Zhangzhou, Fujian province, China; 3 School of Mathematics and Statistics, Henan University, Kaifeng, Henan Province, China; 4 Faculty of Business and Economics, Hong Kong University, Hong Kong, China; 5 Institute for Mathematical Sciences, Renmin University of China, Beijing, China; University of Sao Paulo, BRAZIL

## Abstract

The dissemination of information on networks involves many important practical issues, such as the spread and containment of rumors in social networks, the spread of infectious diseases among the population, commercial propaganda and promotion, the expansion of political influence and so on. One of the most important problems is the influence-maximization problem which is to find out *k* most influential nodes under a certain propagate mechanism. Since the problem was proposed in 2001, many works have focused on maximizing the influence in a single network. It is a NP-hard problem and the state-of-art algorithm IMM proposed by Youze Tang et al. achieves a ratio of 63.2% of the optimum with nearly linear time complexity. In recent years, there have been some works of maximizing influence on multilayer networks, either in the situation of single or multiple influences. But most of them study seed selection strategies to maximize their own influence from the perspective of participants. In fact, the problem from the perspective of network owners is also worthy of attention. Since network participants have not had access to all information of the network for reasons such as privacy protection and corporate interests, they may have access to only part of the social network. The owners of networks can get the whole picture of the networks, and they need not only to maximize the overall influence, but also to consider allocating seeds to their customers fairly, i.e., the Fair Seed Allocation (FSA) problem. As far as we know, FSA problem has been studied on a single network, but not on multilayer networks yet. From the perspective of network owners, we propose a multiple-influence diffusion model MMIC on multilayer networks and its FSA problem. Two solutions of FSA problem are given in this paper, and we prove theoretically that our seed allocation schemes are greedy. Subsequent experiments also validate the effectiveness of our approaches.

## Introduction

There are all kinds of complex networks in our life, such as social networks, the Internet of things, biological networks. The dynamic transmission of information in these networks is closely related to the networks’ own topologies. Network scientists try to explain the dynamics of network by studying the spread of computer and mobile phone viruses [1][2], epidemic diseases [3], rumors [4] and so on. The authors attempt to understand all aspects of network dynamics, such as: the main body of spreading, the pattern of spreading, the carrier of spreading, the efficiency of spreading, and so on. In this paper, we focus on the spreading efficiency of nodes. We strive to find the most influential nodes in multilayer networks and allocate them to the networks’ customers. Next, we will introduce some related works in three categories: 1. Influence maximization in a single network, 2. Influence maximization in multilayer networks, and 3. Fair seed allocation problem.

### Influence maximization in a single network

In recent years, with the rise of social media on the Internet, the communication between people has been enriched. Many social activities that used to be face-to-face are now available online, such as holding conferences, making friends, learning and counseling, etc. The growing demands for the content and form of social activities have led to the emergence of a large number of online social networks. More and more people like to express opinions and share information on online social networks such as Weibo, Facebook and Twitter. In these social networks, information is spread in the form of ‘word-of-mouth’. Under this spreading mechanism, it is an important problem to find the most influential nodes in the social networks. Domingos et al. [5] first put forward the influence-maximization problem which is to find *k* most influential nodes in a social network, that is:

$$\begin{array}{c} \underset{S\in V,|S|=k}{\mathrm{argmax}}\sigma \left(S\right) \end{array}$$ (1)

where *σ* is the influence spread of *k* seeds in *S* and *V* is the node set.

David Kempe et al. [6] proposed two basic models: independent cascade (IC) model and linear threshold (LT) model to describe the influence diffusion mechanism in a single network. However, both models are too simple to precisely reflect the diffusion mechanism on real social networks. To make up for this deficiency, many scholars focus on the improvement and modification of the models. Tim Carnes et al. [7] proposed two models for social network with competitive influences, and dealt with the influence-maximization problem from the follower’s perspective. Budak C. et al. [8] proposed a Model named MCIC and studied the problem of the limitation of ‘bad’ influence. More models of multiple influences competing in a network are considered by researchers [9–14]. Furthermore, sale strategies for virus marketing are also studied [15]. Most previous works focused on the issue of influence propagation within a single network in the past decade. Since the influence-maximization problem is NP-hard [6], so far there is no algorithm of polynomial time complexity to solve the problem. All the algorithms proposed in the previous works are approximate algorithms, they focus on achieving an equilibrium between degree of approximation and time complexity of their algorithms. The state-of-art algorithm IMM [16] has approximately linear time complexity with theoretical guarantee of approximation.

Besides IC and LT model, there are some other network diffusion models, such as the Susceptible-Infectious-Susceptible model (SIS) [17, 18], the Susceptible-Infectious-Recovered model (SIR) [19, 20], the voter model (VM) [3] and the contact process (CP) [3], etc. However, IC and LT are more widely used in the study of influence maximization. The diffusion model used in this paper is IC model.

### Influence maximization in multilayer networks

**Fig 1:**
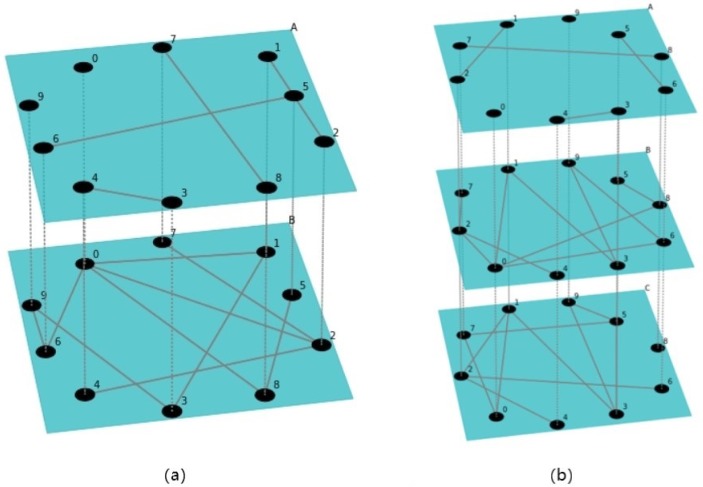
Two examples of multilayer networks. (a) has two layers: A and B. (b) has three layers A, B and C. Node A1 and node B1 are two representatives of entity ‘1’, they are connected by a cross-layer edge (dash line).

Nowadays people act as entities in multiple social networks. It is normal for people to communicate and disseminate information in these networks synchronously. Therefore, compared with a single network, the influence-maximization problem in multilayer networks composed of multiple networks is more worth being studied. Fig 1 is an example of multilayer networks. Nodes marked with the same number represent the account of the same user in each layer of the network. Each user is called an entity, its account in each layer is called a representative of the entity.

In recent years, there have been successive works of maximizing influence in multilayer networks. Ibrahima Gaye et al. [21] proposed a centralized measurement method called ‘Multi-Diffusion Degree’ to select seeds in multilayer networks to maximize the influence. Li Guoliang et al. [22] used the maximum propagation path to approximate the influence between nodes and obtained several solutions of influence-maximization problem of multilayer networks. However, they did not introduce multiple influences into their models. The fact of the matter is that there is often not only one kind of influence spreading in real-world multilayer networks. For example, commercial advertising, the spread of public opinions, rumors and their suppression are all competing influences. Therefore, it is more meaningful to study the diffusion mechanism of multiple influences than that of single influence.

There are other works [23] on diffusion models of multilayer networks. They are mainly concerned with the spread of diseases, opinions and information among the population and how they can interact with each other. Ting Liu et al. [24] constructed a bilayer network, one is the contact network of epidemic spreading which adopts the Susceptible-Infected-Recovered (SIR) model to depict its spreading process, the other is the network of disease information which adopts IC model to depict its spreading process. Velásquez-Rojas et al. [3] proposed the voter model (VM) and the contact process model (CP) to simulate the information propagation network and the epidemic spreading network respectively, and studied the interaction between the two models. Ken T. D. Eames [25] modeled a bilayer network containing a social network of parents and an epidemic infection network of children to study the influence of parents’ social ties on whether children were vaccinated. In addition, there are some papers about bilayer networks using combinations of these diffusion models: Random Walk [26], Kuramoto [26], Voter [3], Contact [3], LACS [27] and GACS [28]. The above works are all aimed at the bilayer networks, and only one kind of information is spread in each layer. What they are concerned with is not finding the most influential nodes, but the impact of propagation on the whole network from some arbitrary nodes. Unlike them, our work is not limited to the bilayer networks. The number of influences can be arbitrary and they can be freely spread across layers, and what we concern is to find the *k* most influential nodes and allocate them fairly.

### Fair seed allocation in a single network and multilayer networks

Whether for a single network or multilayer networks, the previous studies [9, 10, 11, 12, 13, 14] have considered multiple influences, but mostly from the perspective of participants, that is to find the optimal seeds strategy to maximize its own influence on the premise that the opponents have already chosen their seeds. However, participants only have local information of the network, so it is more reasonable to study problems using global information from the perspective of network owners. By selling seeds to its customers, the network platform provides viral marketing services. The network platform not only considers the selection of the most influential seeds, but also considers the equilibrium of seeds allocation to different customers according to their budgets. Fair Seed Allocation (FSA) problem originates from the problem of how to distribute seeds for the network platform which carries out viral marketing. With the in-depth study of the problem of maximizing influence on single network and multilayer networks, it is realized that we should not only consider the maximization of influence, but also consider how to rationally distribute seeds to the customers involved in viral marketing from the perspective of network platform owners in order to maximize the customers’ expectations. That is to say, the network platform should not only find the most influential seeds, but also distribute them reasonably to its customers to make them satisfied. FSA is first introduced by Wei Lu et al. [29] under their ‘K-LT’ model.

Let budgets of customers be γ_1_, γ_2_, …, γ_*t*_, total budget be $\gamma =\Sigma_{i=1}^{t}\gamma_{i}$, and unit price be a constant *F*. Fair seed allocation problem is to find a total seed set S of minimum size *k*, s.t. σ(S)≥F*γ, and a partition of $S=\{S_{1},...,S_{t}\}$ to maximize fairness *f*:

$$\begin{array}{c} {\mathrm{argmax}}_{S}\left(f=\frac{\min_{i=1}^{t}\frac{\sigma (S_{i},S)}{\gamma_{i}}}{\max_{i=1}^{t}\frac{\sigma (S_{i},S)}{\gamma_{i}}}\right), \end{array}$$ (2)

where $\sigma (S_{i},S)$ is the influence spread of *S*_*i*_ when S is the total seed set.

Ying Yu et al. [30] discussed Fair Seed Allocation problem under their diffusion model ‘TIC’. However, they are only studying FSA problem on a single network. Large Internet companies often have more than one online social network, and almost everyone is active in more than one social network. Finding the most influential seeds in multilayer networks and allocating them to their customers in a balanced way becomes an urgent need to be solved. In this work, we concern FSA problem on multilayer networks. FSA has two phases. The first phase is to find the *k* most influential seeds and the second phase is to allocate these seeds fairly to all customers.

## MMIC model and the influence-maximization problem

In this section, we consider the first phase of FSA, i.e., find the *k* most influential seeds in multilayer networks. At first, we will propose a diffusion model of multiple influences competing in multilayer networks, called MMIC.

### MMIC model

In a multilayer network, the total seed set is denoted by S. {*S*_1_, *S*_2_, …, *S*_*t*_} is a partition of S. Elements in *S*_*i*_ are seeds of influence *i*, where *i* = 1, 2, …, *t*. We call influence *i* **color** *i* for convenience, i.e., there are *t* colors of seeds. The status of a node is either inactive or active. Initially *S*_*i*_ is a set of *i*-color seeds, in which each node is active with color *i*, *i* = 1, 2, …, *t* and all non seeds are inactive. When a node *u* is active with color *i*, it will attempts to activate its non-seed out-neighbor *v* with color *i*. Each activation attempts only once with a success probability respectively, and each node can receive *i*-activation more than once (*i* = 1, 2, …*t*), and this inactive-to-active transfer is irreversible. The propagation goes on until there are no more activations. An entity is activated when one of its representatives is active. When the activation process ends, each active entity *v* decides to a color *i* with probability $P_{v}^{i}$, where $P_{v}^{i}$ is the proportion of *i*-color activations and all activations received by entity *v*. In particular, for each entity who has seeds as its representatives decides to color *i* with a probability of the proportion of the *i*-color seeds among its colored representatives. This diffusion model is called **MMIC**. The **influence spread of** *S*_*i*_ is the expected number of *i*-color entities, denoted by $\sigma (S_{i},S)$. The **total influence spread** is the expected number of colored entities, denoted by σ(S).

It is easy to verify that $\sigma \left(S\right)=\Sigma_{i=1}^{t}\sigma \left(S_{i},S\right)$. Since each active entity has to decide to a color finally, assigning all seeds to a uniform color does not change the total influence spread of S.

**Fig 2:**
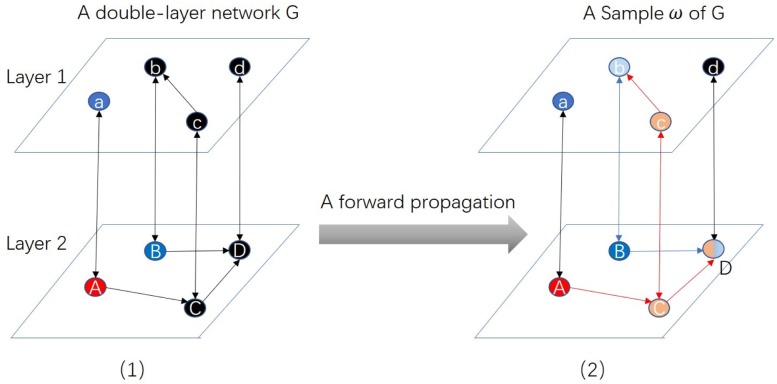
An example of one propagation of multiple influences (colors) in a multilayer network *G*. (1) is a double-layer network *G*. The uppercase and lowercase of one letter represents one entity, uppercase and lowercase nodes are the representatives of the entity in two layers respectively. Red node ‘A’, blue node ‘a’ and blue node ‘B’ are three seeds of two influences respectively. (2) is an outcome of *G* under MMIC, i.e. a sample *ω*. Pink and pale blue nodes are the nodes who have received red and blue activations, respectively. Because entity ‘Aa’ has one representative ‘A’ as a red seed, and one representative ‘a’ as a blue seed, entity ‘Aa’ decides to each color with a probability of 1/2. Entity ‘Bb’ only owns blue seed as its representative, so ‘Bb’ ultimately decides to blue. Entity ‘Cc’ only receives red activations, so ‘Cc’ ultimately decides to red. Entity Dd receives red activation once and blue activation once. Therefore, entity Dd decides to each color with a probability of 1/2.

Fig 2 gives an example of one propagation of multiple influences, i.e., colors in a multilayer network *G*. Red and blue nodes are seeds, pink and pale blue nodes are nodes activated by them respectively. Node D receives two types of activation. In the end, the entity ‘Dd’ can only decide to one color, i.e., red or blue.

Now we study how to calculate the influence spread of each color *i*. For each edge *e* in network *G*, we delete it with probability 1 − *p*(*e*). All of the possible outcomes of this process with their probabilities constitute a probability space Ω. Each element of Ω is called a **sample** from Ω, denoted by *ω*. For a given sample *ω*, a fixed set *S* ⊆ *V* and a given entity *v*_*j*_, if there is a path *τ* from *S* to one of the representatives of entity *v*_*j*_, we call that *v*_*j*_ is **reachable** from *S*, and the path *τ* is called a *i*-**color live path** from *S* to *v*_*j*_. Each edge of path *τ* is called a *i*-**color live edge**. For example, ‘A-C-D’ is a red live path, or we say that entity ‘Dd’ is reachable from *S* = {*A*, *C*, *D*} in Fig 2(2).

**Fig 3:**
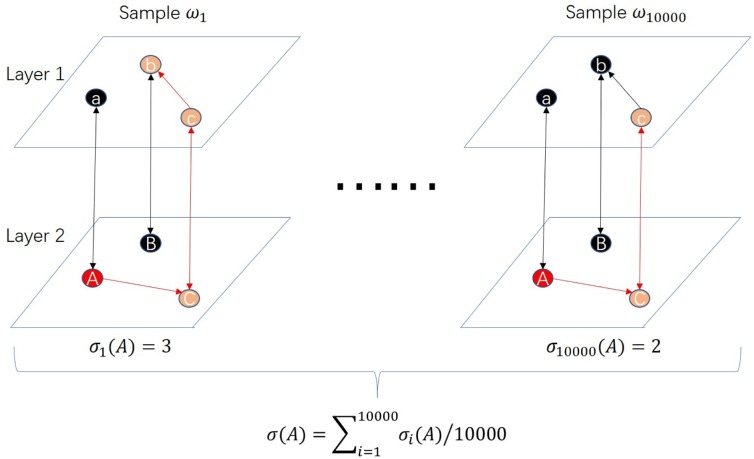
An example of calculating influence spread using Monte Carlo method. The 10000 samples are the outcomes of 10000 propagations of *G* under MMIC model respectively. The red node is the seed *A*. The pink nodes are the active nodes in each sample. The dark nodes are the inactive nodes. The red edges are the live edges. *σ*_*i*_(*A*) is the number of active entities in sample *i*. The influence spread of node *A* is the average of all *σ*_*i*_(*A*)(where *i* = 1, 2, …, 10000).

A live path from *S* to *v*_*j*_ indicates that seed set *S* can activate entity *v*_*j*_. Let $h_{j}^{\omega }\left(S\right)$ be the indicator function of that event. In other words, $h_{j}^{\omega }\left(S\right)=1$ when *v*_*j*_ is reachable from *S*, otherwise $h_{j}^{\omega }\left(S\right)=0$. It can be verified the influence spread of *S* is: $\sigma \left(S\right)=\sum Pro\left(\omega \right)\sum_{i=1}^{n}h_{j}^{\omega }\left(S\right)$. However, this formula of influence spread is not practical because the probability of the sample *Pro*(*ω*) is difficult to calculate. We use Monte Carlo method to calculate the influence spread *σ*(*S*). Fig 3 gives an example of calculating the influence spread of seed set *S*. The red nodes A is the seed, i.e., *S* = {*A*}. The pink nodes are the nodes activated by the seed. Each subfigure is the outcome of one propagation. We set the number of iterations to be 10000. The influence spread of the seed is equal to the average number of activated nodes.

We declare that network *G* appear in the rest of the paper is multilayer network *G*. Many other notations used frequently in this paper are listed in Table 1.

**Table 1:** Notations.

*G*(*V*, *E*, *W*)	multilayer network *G* with node set *V*, edge set *E*, and weight set *W*
*v*_*j*_, *n* and *N*	the *j*th entity of *G*, the number of entities and the entity set
*σ*(*S*)	the influence spread of *S*
Ω	the probability space of outcomes generated by deleting every edge *e* with probability 1 − *P*(*e*)
*ω*	a sample from Ω, an outcome of one propagation.
*τ*	a live path
*RRE*	reverse reachable set for entity

### The influence-maximization problem of MMIC

The first step of FSA problem is seeking *k* most influential seeds in *G*. So we first consider that all seeds belong to the same color. The influence-maximization problem of IC model is a special case of the problem of MMIC when the weight of every cross-layer edge is 1 or the number of layers is 1. Since the influence-maximization problem of IC model is NP-hard, so is of MMIC. Therefore, there is no polynomial time complexity algorithm to solve this problem. According to [6], if *σ*(*S*) is monotone and submodular w.r.t. *S*, there is a greedy scheme of seed selection which can provide a (1 − 1/*e*)-approximation. Although it can be proved that the influence function *σ*(*S*) under MMIC model is monotone and submodular w.r.t. *S*, the time complexity of Algorithm 1 (Greedy) is still high, and it is not suitable for large multilayer networks.

**Algorithm 1** Greedy

1: *S*′ ← *ϕ*

2: **for** *i* = 1 to *k* **do**

3:  $u={\mathrm{argmax}}_{u\in V\backslash S^{\prime }}\left(\sigma \left(S^{\prime }⋃\left\{u\right\}\right)-\sigma \left(S^{\prime }\right)\right)$;

4:  **if** *σ*(*S*′⋃{*u*}) − *σ*(*S*′) > 0 **then**

5:   *S*′ ← *S*′⋃{*u*}

6:  **end if**

7: **end for**

8: **return** *S*′

Intuitively, nodes with large out-degree are generally influential. Based on this idea, we use the following method to select seeds. First we select the node with largest out-degree, and then delete it from the network. After repeating this process *k* times we have *k* seeds, see Algorithm 2.

**Algorithm 2** Degree

1: *S*′′ ← *ϕ*

2: *G*′ = *G*

3: **for** *i* = 1 to *k* **do**

4:  $u={\mathrm{argmax}}_{u\in G^{\prime }}outdegree(u)$;

5:  *S*′′ ← *S*′′⋃{*u*}

6:  *G*′ = *G*′ − *u*

7: **end for**

8: **return** *S*′′

The advantage of the degree method is that the computing speed is very fast, but there is no theoretical guarantee of its performance, i.e., its performance varies with different networks.

Youze Tang et al. [16] proposed an algorithm IMM to solve the influence-maximization problem on a single network. It is the state-of-art algorithm we have known which has both guarantee of approximation and nearly linear time complexity. The core idea of IMM is based on the concept of reverse reachable (RR) set. A RR set of node *u* consists of all nodes from which *u* is reachable in sample *ω*, i.e., it contains all nodes can directly or indirectly activate node *u* in sample *ω*. Furthermore, if node *u* is chosen uniformly at random from *V*, a RR set is called a random RR set. Suppose we have *θ* (large enough, for example, *θ* = 10000) random RR sets from *θ* random nodes. If a node *x* appears 9999 times in these 10000 RR sets, we say that node *x* overlaps 9999 RR sets. From the definition of RR set, we can infer that *x* has great influence spread because it has the ability to activate many of these 10000 nodes as a seed. For a given node set *S*, that *S* overlaps more random RR sets indicates that *S* is capable of activating more nodes as the seed set. Therefore the influence-maximization problem of IC model is transferred to seeking for a node set *S* of size *k* to overlap the most random RR sets. For our MMIC model, the influence spread is the expected number of final active entities instead of nodes, so we need to define reverse reachable set for entity.

Let *v*_*j*_ be an entity of multilayer network *G*, and *ω* be a sample from Ω. Let $R_{j}^{\omega }$ be the set consists of all nodes from which *v*_*j*_ can be reachable. The set $R_{j}^{\omega }$ is called the **reverse reachable set of entity** *v*_*j*_, **RRE** for short. If *v*_*j*_ is chosen uniformly at random from *N*, and *ω* is a random outcome of MMIC, the RRE is called a random RRE.

**Fig 4:**
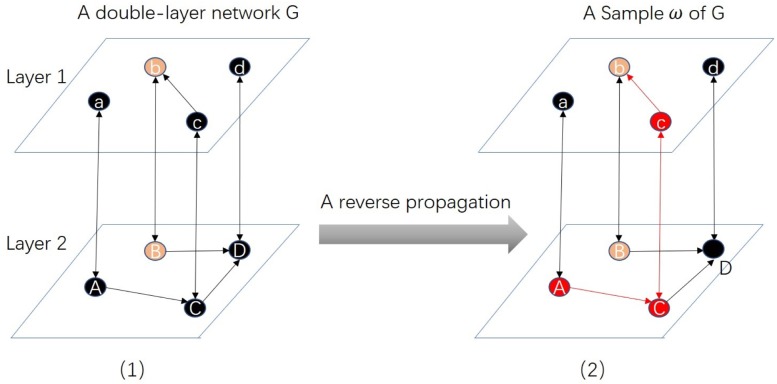
An example of random RRE of entity Bb in a multilayer network. (1) is a bilayer network *G*, the pink nodes ‘B’ and ‘b’ are representatives of entity Bb. After a reverse propagation (along the opposite direction of edges) from ‘B’ and ‘b’, we have a sample *ω*, i.e., (2). The active nodes (B,b,c,C,A) which constitute a random RRE of entity Bb.

Fig 4 shows an example of random RRE of entity ‘Bb’ in a multilayer network. After a random reverse propagation (along the opposite direction of the edges) from B and b (the pink nodes), we have the outcome of active nodes (the red and pink nodes): *R*_1_ = {A,B,C,b,c} which is a random RRE of entity ‘Bb’.

**Fig 5:**
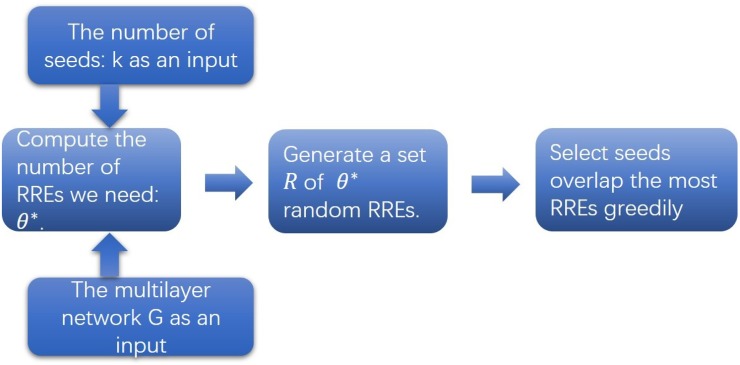
The procedure of RRE method (Algorithm 3).

Fig 5 shows the procedure of RRE method. Firstly, we calculate the number of random RREs we needed: θ* (The value of θ* is calculated by algorithm IMM from [16]). Secondly, we generate θ* random RREs. At last, we add nodes that overlap the most random RREs as seeds greedily. RRE method of influence-maximization problem under MMIC model is presented as Algorithm 3.

**Algorithm 3** RRE (θ*, *R*)

1: Compute θ* and *R* from algorithm 2 in [16]

2: **while** |*R*|≤θ* **do**

3:  Select an entity *v*_*j*_ from *G* uniformly at random;

4:  Generate a random RRE of *v*_*j*_ and insert it into *R*;

5: **end while**

6: S* ← *ϕ*

7: **for** *i* = 1 to *k* **do**

8:  $u={\mathrm{argmax}}_{u}\left(F_{R}\left(S^{*}⋃\left\{u\right\}\right)-F_{R}\left(S^{*}\right)\right)$;

9:  S* ← *S*⋃{*u*}

10: **end for**

11: **return** S*

Our Algorithm 3 extends the RR set of the algorithm 2 in [16] to RRE, which does not change the time complexity, so according to [16], the time complexity of our Algorithm 3 is still $O(\frac{(k+l)(n+m)\text{log}\;n}{{∊}^{2}})$, where *n* and *m* are the numbers of nodes and edges, respectively in the multilayer networks, *k* is the number of seeds, The two parameters *l* and *ϵ* are set to the same value as in [16]. Next, we will demonstrate the effectiveness and robustness of RRE method relative to degree method through simulation.

#### Simulation

*Experimental setup*. We use ‘Stanford Large Network Dataset Collection’ from [31]. Two real social networks are selected: Wikipedia vote network and Bitcoin Alpha trust weighted signed network. Wikipedia is a free encyclopedia written by volunteers from all over the world. Anyone who wants to participate in managing the site needs to submit an application to the Wikipedia community for public discussion and voting. Wiki-Vote network contains all the voting data of Wikipedia from the inception of Wikipedia to January 2008. The nodes in the network Wiki-Vote represent Wikipedia users, and the directed edges from node *i* to node *j* indicate that user *i* voted for user *j*. Bitcoin Alpha is a Bitcoin trading platform. Since Bitcoin transactions are anonymous, users need to maintain their reputation to prevent transactions with users at risk of fraud. Each user grades the others from -10 (total distrust) to +10 (total trust). The nodes represent the users, the edges represent the trust relationships between them, which constitute the network Bitcoin-alpha. By randomly deleting ten percent of the edges of Wiki-Vote respectively, we get two networks named Wiki-Vote1 and Wiki-Vote2. After adding cross-layer edges between them, we get multilayer network Wiki-Vote0. Network Bitcoin-alpha0 is also constructed in the same way. The way to assign weights is WC. That is, the weight of edge (*u*, *v*) is the reciprocal of *v*’s in-degree. WC is one of the most commonly used weighting methods because it is a reasonable normalized measure. That is to say, when we do not know the probabilities of node *u* activating its out-neighbors, a reasonable way to define these probabilities is to assume the probabilities are all the same, and the sum of them is 1. The basic statistics of the datasets are shown in Table 2.

**Table 2:** Network info.

Network	#Nodes	#Edges	Average in-degree	Average out-degree
Wiki-Vote	7,115	103,689	14.5733	14.5733
Bitcoin-alpha	3,783	24,186	6.3933	6.3933
Wiki-Vote0	14,172	200,814	14.1698	14.1698
Bitcoin-alpha0	7,564	51100	6.7557	6.7557

Here is our lab server configuration. Operating system: CentOS; CPU: Intel (R) Xeon (R) E5, 32 core; Memory 64G.

*Evaluation method*. Our evaluation index is the influence spread of selected *k* seeds by different methods. We do experiments on network Wiki-Vote0 and Bitcoin-alpha0 with three methods: random, degree and RRE. Because the time complexity of greedy algorithm is too high, we do not use it in experiments.

**Fig 6:**
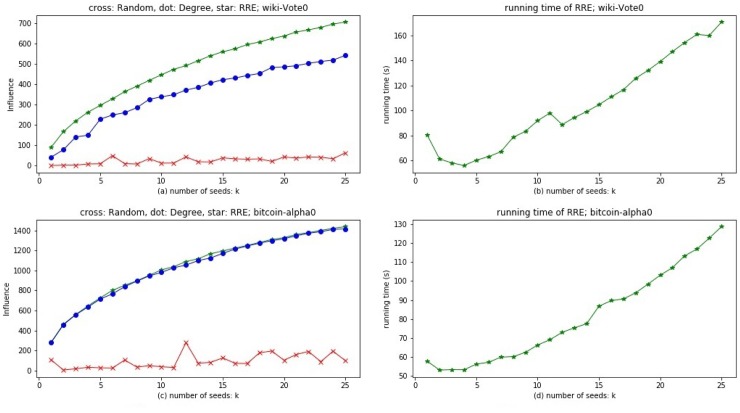
The influence spread of three methods and the running time of RRE method. The number of iterations to calculate influence spread is 10000. For (a) and (c), abscissa is the number of seeds; ordinates represent the influence spread; red cross, blue dot and green star line represent the methods of random, degree and RRE respectively. For (b) and (d), abscissa is the number of seeds, ordinates represent the running time (s) of RRE method.

The experimental results of influence maximization are shown in Fig 6. Nodes with large out degrees often have great influence spread, so the degree method can sometimes achieve good results, such as the results on Bitcoin-alpha0, see Fig 6(c). But for some multilayer networks, such as the results on Wiki-Vote0, see Fig 6(a), the degree method is not very effective. The reason is that it ignores nodes with small degree but associated with important nodes. RRE method is more robust than degree method because it can reach nearly (1 − 1/*e*) of the optimal influence spread in the worst case for any multilayer networks. From Fig 6, we can see that RRE method outperforms others on both Wiki-Vote0 and Bitcoin-alpha0. Considering time complexity and robustness, we use RRE method to select the *k* most influential seeds for FSA problem.

## Fair seed allocation problem of MMIC model

After finding the *k* most influential seeds by RRE method, we consider the issue of fairly allocating the *k* seeds to *t* different customers (colors) from the perspective of the platform owner, which is the second phase of FSA. That is to say, make sure that for each color, the expected influence spread is proportional to its budget.

Now we allocate *k* seeds to *t* different colors to maximize *f* in Eq (2). Before that, some preparatory knowledge should be given first.

Given a sample *ω*, let *a*^*ω*^(*S*_*i*_, *v*) be the number of *i*-color live paths from *i*-color seed set *S*_*i*_ to entity *v*. Let $b^{\omega }\left(S_{i},S,v\right)$ be the probability of that entity *v* is *i*-colored as *S*_*i*_ is the *i*-color seed set and S is the total seed set. Let $c_{S_{i}}$ be the number of representatives of *v* in *S*_*i*_ and *c* be the number of representatives of *v* in S. Let *N*_1_ be the set of entities who has at least one seed as their representative. Then we have:

$$\begin{array}{c} b^{\omega }\left(S_{i},S,v\right)=\{\begin{array}{cc} \frac{a^{\omega }\left(S_{i},v\right)}{a^{\omega }\left(S,v\right)}, & if\;v\notin N_{1}.\\ \frac{c_{S_{i}}}{c}, & if\;v\in N_{1}. \end{array} \end{array}$$ (3)

If $a^{\omega }\left(S,v\right)=0$, i.e., there is no live path from S to *v*, we specify that $b^{\omega }\left(S_{i},S,v\right)=0$.

**Theorem 1**.

$$\begin{array}{c} \sigma \left(S_{i},S\right)=\Sigma_{s\in S_{i}}\sigma \left(s,S\right) \end{array}$$ (4)

*Proof*.

$$\begin{array}{c} \sigma \left(S_{i},S\right)\\ =\Sigma_{\omega \in \Omega }Pro\left(\omega \right)\sigma^{\omega }\left(S_{i},S\right)\\ =\Sigma_{\omega \in \Omega }Pro\left(\omega \right)\Sigma_{v\in N}b^{\omega }\left(S_{i},S,v\right)\\ =\Sigma_{\omega \in \Omega }Pro\left(\omega \right)\left[\Sigma_{v\in N\backslash N_{1}}\frac{a^{\omega }\left(S_{i},v\right)}{a^{\omega }\left(S,v\right)}+\Sigma_{v\in N_{1}}\frac{c_{S_{i}}}{c}\right]\\ =\Sigma_{\omega \in \Omega }Pro\left(\omega \right)\Sigma_{v\in N\backslash N_{1}}\frac{a^{\omega }\left(S_{i},v\right)}{a^{\omega }\left(S,v\right)}+\Sigma_{\omega \in \Omega }Pro\left(\omega \right)\Sigma_{v\in N_{1}}\frac{c_{S_{i}}}{c}\\ =\Sigma_{\omega \in \Omega }Pro\left(\omega \right)\Sigma_{v\in N\backslash N_{1}}\frac{a^{\omega }\left(S_{i},v\right)}{a^{\omega }\left(S,v\right)}+\Sigma_{v\in N_{1}}\frac{c_{S_{i}}}{c}\\ =\Sigma_{\omega \in \Omega }Pro\left(\omega \right)\Sigma_{v\in N\backslash N_{1}}\Sigma_{s\in S_{i}}\frac{a^{\omega }\left(s,v\right)}{a^{\omega }\left(S,v\right)}+\Sigma_{v\in N_{1}}\frac{\Sigma_{s\in S_{i}}c_{s}}{c}\\ =\Sigma_{s\in S_{i}}\left[\Sigma_{\omega \in \Omega }Pro\left(\omega \right)\Sigma_{v\in N\backslash N_{1}}\frac{a^{\omega }\left(s,v\right)}{a^{\omega }\left(S,v\right)}+\Sigma_{v\in N_{1}}\frac{c_{s}}{c}\right]\\ =\Sigma_{s\in S_{i}}\Sigma_{\omega \in \Omega }Pro\left(\omega \right)\left[\Sigma_{v\in N\backslash N_{1}}\frac{a^{\omega }\left(s,v\right)}{a^{\omega }\left(S,v\right)}+\Sigma_{v\in N_{1}}\frac{c_{s}}{c}\right]\\ =\Sigma_{s\in S_{i}}\Sigma_{\omega \in \Omega }Pro\left(\omega \right)\sigma^{\omega }\left(s,S\right)\\ =\Sigma_{s\in S_{i}}\sigma \left(s,S\right) \end{array}$$

Recall that $\sigma \left(S\right)=\Sigma_{i=1}^{t}\sigma \left(S_{i},S\right)$, then we have $\sigma \left(S\right)=\Sigma_{i=1}^{t}\Sigma_{s\in S_{i}}\sigma \left(s,S\right)$. That means the influence spread of *S*_*i*_ is the sum of influence spread of every element *s* in *S*_*i*_ while *s* is the only *i*-seed in S, and the total influence spread is the sum of influence spread of every element *s* in S while *s* is the only *i*-seed in S.

**Theorem 2**. $\sigma (S_{i},S)$ *is monotone and submodular w.r.t. S*_*i*_.

*Proof*. By the proof of Theorem 1,

$$\sigma (S_{i},S)=\Sigma_{\omega \in \Omega }Pro\left(\omega \right)\left[\Sigma_{v\in N\backslash N_{1}}\frac{a^{\omega }\left(S_{i},v\right)}{a^{\omega }\left(S,v\right)}+\Sigma_{v\in N_{1}}\frac{c_{S_{i}}}{c}\right].$$

Since $Pro\left(\omega \right),a^{\omega }\left(S,v\right)$ and *c* are all non-negative and independent of *S*_*i*_, we have that $\sigma (S_{i},S)$ is a non-negative linear combination of *a*^*ω*^(*S*_*i*_, *v*) and $c_{S_{i}}$. All we need to do is to prove that *a*^*ω*^(*S*_*i*_, *v*) and $c_{S_{i}}$ are both monotone and submodular w.r.t. *S*_*i*_.

(i) Monotonicity
  Given a sample *ω*, for arbitrary *x* ∈ *V*, the number of *i*-color live paths from *i*-color seed set *S*_*i*_⋃{*x*} to entity *v* is *a*^*ω*^(*S*_*i*_⋃{*x*}, *v*) = *a*^*ω*^(*S*_*i*_, *v*)+ *a*^*ω*^(*x*, *v*). Therefore *a*^*ω*^(*S*_*i*_⋃{*x*}, *v*) − *a*^*ω*^(*S*_*i*_, *v*) = *a*^*ω*^(*x*, *v*)≥0. *a*^*ω*^(*S*_*i*_, *v*) is monotone w.r.t. *S*_*i*_.
  Similarly, $c_{S_{i}⋃\left\{x\right\}}-c_{S_{i}}=c_{x}\geq 0$, then $c_{S_{i}}$ is monotone w.r.t. *S*_*i*_.
(ii) Submodularity
  For arbitrary *x* ∈ *V* and $S_{i}\subseteq S_{i}^{\prime }\subseteq V$, there are two cases:
  (Case 1) If $x\in S_{i}^{\prime }$, $a^{\omega }\left(S_{i}^{\prime }⋃\left\{x\right\},v\right)-a^{\omega }\left(S_{i}^{\prime },v\right)=0$. Then we have $a^{\omega }\left(S_{i}⋃\left\{x\right\},v\right)-a^{\omega }\left(S_{i},v\right)\geq a^{\omega }\left(S_{i}^{\prime }⋃\left\{x\right\},v\right)-a^{\omega }\left(S_{i}^{\prime },v\right)=0$.
  (Case 2) If $x\notin S_{i}^{\prime }$, we have $a^{\omega }\left(S_{i}^{\prime }⋃\left\{x\right\},v\right)-a^{\omega }\left(S_{i}^{\prime },v\right)=a^{\omega }\left(x,v\right)=a^{\omega }\left(S_{i}⋃\left\{x\right\},v\right)-a^{\omega }\left(S_{i},v\right)$.
  Therefore, *a*^*ω*^(*S*_*i*_, *v*) is submodular w.r.t. *S*_*i*_.
  Similarly, it is easy to verify that $c_{S_{i}}$ is submodular w.r.t. *S*_*i*_.
  In summary, $\sigma (S_{i},S)$ is monotone and submodular w.r.t. *S*_*i*_.

By Theorem 1 and 2, the seed allocation phase of FSA problem is demonstrated as follows:

Firstly, compute all σ(s,S) for any s∈S,j=1,2,...,k. Note that S is the set of *k* seeds from Algorithm 3. Secondly, allocate all *k* elements in S to *t* seed sets *S*_1_, *S*_2_, …, *S*_*t*_ to make the differences among $f_{i}=\frac{\Sigma_{s\in S_{i}}\sigma \left(s,S\right)}{\gamma_{i}},i=1,2,..,t$ minimized. In other words, maximizing $f=\frac{\min_{i=1}^{t}f_{i}}{\max_{i=1}^{t}f_{i}}$, where *f*_*i*_ and *f* are defined in Eq (2). We are going to propose two greedy methods for seed allocation which are guaranteed by the following theorem:

**Theorem 3**. *Assume that* S *is the total seed set obtained in the first phase of FSA problem. Allocating* s∈S *one by one to i-color seed set S*_*i*_ *who has the current least f*_*i*_ *is the greedy choice of maximizing f*.

*Proof*. Now we have to prove that allocating seed *s* to *i*-color who has the current least *f*_*i*_ is the greedy choice of maximizing *f*. In other words, it is locally optimal in each step of allocation. We use reduction to absurdity to prove that.

Without losing generality, assume that *f*_1_ ≤ *f*_2_ ≤ … ≤ *f*_*t*_ currently, but we allocate seed *s* to *j*-color, *j* ≠ 1. Let $f_{j}^{\prime }$ be the updated value of *f*_*j*_ when the allocation has been done, while the other *f*_*i*_, *i* ≠ *j* remain the same according to Theorem 1.

(Case 1) If $f_{j}^{\prime }\leq f_{t}$, the minimum and maximum of *f*_*i*_, *i* = 1, 2, …, *t* is not changed. Therefore, we have the updated value of *f* as $f^{\prime }=\frac{f_{1}}{f_{t}}$. However, the updated value of *f* would be $f^{\prime \prime }=\frac{\min\{f_{1}^{\prime },f_{2}\}}{f_{t}}$ if *s* is allocated to color 1. By Theorem 1 and 2, *f*′ < *f*′′, then allocating *s* to color *j* is not the greedy choice.

(Case 2) If $f_{j}^{\prime }>f_{t}$, we have the updated value of *f* as $f^{\prime }=\frac{f_{1}}{f_{j}^{\prime }}$. If *s* is allocated to color 1, there are two subcases.

(Subcase 1) If $f_{1}^{\prime }\leq f_{t}$, we have $f^{\prime \prime }=\frac{\min\{f_{1}^{\prime },f_{2}\}}{f_{t}}$ as the updated value of *f* if *s* is allocated to color 1. By Theorem 1 and 2, $f_{1}\leq \min\left\{f_{1}^{\prime },f_{2}\right\}$ and $f_{j}^{\prime }>f_{t}$, then we have *f*′ < *f*′′.

(Subcase 2) If $f_{1}^{\prime }>f_{t}$, we have $f^{\prime \prime }=\frac{f_{2}}{f_{1}^{\prime }}$ as the updated value of *f* if *s* is allocated to color 1. By Theorem 1 and 2, *f*_1_ ≤ *f*_2_ and $f_{j}^{\prime }>f_{1}^{\prime }$, then we have *f*′ < *f*′′.

In both subcases, allocating *s* to color *j*, *j* ≠ 1 is not the greedy choice. In summary, allocating *s* to color *i* who has the current least *f*_*i*_ is the greedy choice of maximizing *f*.

The orders of seed allocation are specified in a way from [30]. Firstly, we get *k* and S as the output of Algorithm 3. Secondly, we initialize all seed sets *S*_*i*_ to empty sets and initialize all their *f*_*i*_ to 0. At last, we allocate s∈S one by one by its σ(s,S) non-decreasingly into *S*_*i*_ with the current least *f*_*i*_ (by Theorem 3). In accordance with this idea, we propose Algorithm 4 to allocate seeds.

**Algorithm 4** Fair1

1: *k* = min{*k* = |S*|:*σ*(S*) − *F* * *γ* ≥ 0}, where S* is the output of Algorithm 3.

2: $S\leftarrow S^{*}$;

3: *S*_*i*_ ← *ϕ*, *i* = 1, 2, …, *t*;

4: *f*_*i*_ ← 0, *i* = 1, 2, …, *t*;

5: Sort the elements of S into {*s*_1_, *s*_2_, …, *s*_*k*_} by their $\sigma (s_{j},S)$ non-decreasingly;

6: **for** *j* = 1 to *k* **do**

7:  *S*_i*_ ← *S*_i*_⋃{*s*_*j*_}, where i* = arg_1≤*i*≤*t*_min*f*_*i*_;

8:  $f_{i}^{*}=\frac{\sigma (S_{i^{*}},S)}{\gamma_{i^{*}}}$;

9: **end for**

10: **return** {*S*_1_, *S*_2_, …, *S*_*t*_}

Here is an example of how Algorithm 4 and 5 work. We assume there are three colors with budgets γ_1_: γ_2_: γ_3_ = 1: 2: 3, and the number of seeds *k* = 6. Suppose that after the first phase of FSA, we obtain six seeds: *s*_*j*_, *j* = 1, 2, …, 6. with their influence spread $\sigma (s_{j},S)=1,5,6,7,12,13$ for *j* = 1, 2, …, 6 calculated by Algorithm 3.

**Fig 7:**
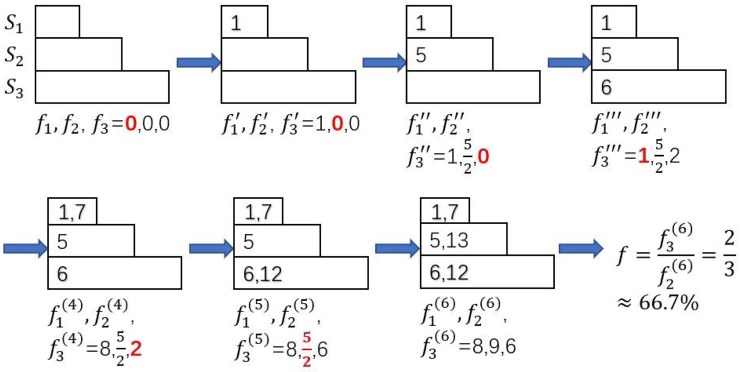
The procedure of Fair1 (Algorithm 4) when budgets are 1:2:3 and $\sigma (s_{i},S)=1,5,6,7,12,13$ for *i* = 1, 2, …, 6.

Now, we use Algorithm 4 for seed allocation phase. The size of the three rectangles in Fig 7 represents the size of the budgets. In this phase, we should allocate the six seeds to three colors (*S*_1_, *S*_2_, *S*_3_) to maximize *f*. We initialize all seed sets *S*_*i*_ to empty sets and set all initial values of *f*_*i*_ to be 0. We allocate *s*_*j*_ one by one (by their $\sigma (s_{j},S)$ non-decreasingly) into *S*_*i*_ who has the least value of *f*_*i*_ (in red) currently, and update the value of *f*_*i*_. The final allocation of seeds is *S*_1_ = {*s*_1_, *s*_4_}, *S*_2_ = {*s*_2_, *s*_6_}, *S*_3_ = {*s*_3_, *s*_5_} and *f* ≈ 66.7%.

Now we will propose another method for the orders of allocations. Firstly we get *k* and S as the output of Algorithm 3. Secondly, we initialize all seed sets *S*_*i*_ to empty sets, and initialize *f*_*i*_ to the reciprocals of its budget. At last, we allocate s∈S one by one by its σ(s,S) non-increasingly into *S*_*i*_ with the current least *f*_*i*_ (by Theorem 3). Therefore, we obtain another method of seed allocation, i.e., Algorithm 5:

**Algorithm 5** Fair2

1: *k* = min{*k* = |S*|:*σ*(S*) − *F* * *γ* ≥ 0}, where S* is the output of Algorithm 3.

2: $S\leftarrow S^{*}$;

3: *S*_*i*_ ← *ϕ*, *i* = 1, 2, …, *t*;

4: $f_{i}\leftarrow \frac{1}{\gamma_{i}},i=1,2,...,t$;

5: Sort the elements of S into {*s*_1_, *s*_2_, …, *s*_*k*_} by their $\sigma (s_{j},S)$ non-increasingly;

6: **for** *j* = 1 to *k* **do**

7:  *S*_i*_ ← *S*_i*_⋃{*s*_*j*_}, where i* = arg_1≤*i*≤*t*_min*f*_*i*_;

8:  $f_{i}^{*}=\frac{\sigma (S_{i^{*}},S)}{\gamma_{i^{*}}}$;

9: **end for**

10: **return** {*S*_1_, *S*_2_, …, *S*_*t*_}

**Fig 8:**
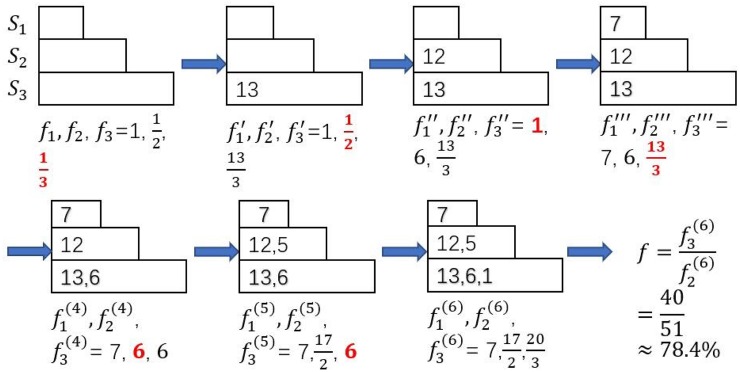
The procedure of Fair2 (Algorithm 5) when budgets are 1:2:3 and $\sigma (s_{i},S)=1,5,6,7,12,13$ for *i* = 1, 2, …, 6.

Now, we use Algorithm 5 for seed allocation phase in stead of Algorithm 4. The process is shown in Fig 8. We set initial value of *f*_*i*_ to be the reciprocal of its budget, that is $\frac{1}{\gamma_{i}}$. We allocate *s*_*j*_ one by one (by their $\sigma (s_{j},S)$ non-increasingly) into *S*_*i*_ who has the least value of *f*_*i*_(in red) currently, and update the value of *f*_*i*_ meanwhile until all six seeds are allocated. The final allocation of seeds is *S*_1_ = {*s*_4_}, *S*_2_ = {*s*_2_, *s*_5_}, *S*_3_ = {*s*_1_, *s*_3_, *s*_6_} and *f* ≈ 78.4%.

**Fig 9:**
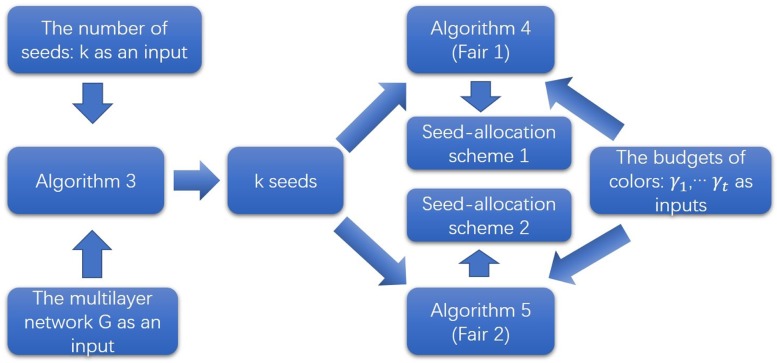
The procedure of solutions of Fair seed allocation problem.

In summary, Fig 9 is a flowchart illustrating our solutions of FSA problem.

### Simulation

Considering that there are different budget configurations in real world, we choose both balanced budgets of 1:1, 1:1:1, 1:1:1:1, and unbalanced budgets of 1:2, 1:2:3, 1:2:3:4. Experiments are conducted on Wiki-Vote0 and Bitcoin-alpha0 respectively. The abscissa is the number of seeds: *k*, the ordinate is fairness *f*. Three methods are considered, Random (red cross line), Fair1 (blue dot line), and Fair2 (green star line). The result of Random method is the average result of 10,000 Monte Carlo simulations. The closer *f* is to 1, the more effective the method is. The time complexity of Algorithm 4 and 5 of seed allocation phase is *O*(k*t), where *k* is the number of seeds and *t* is the number of colors (customers).

### Discussion

**Fig 10:**
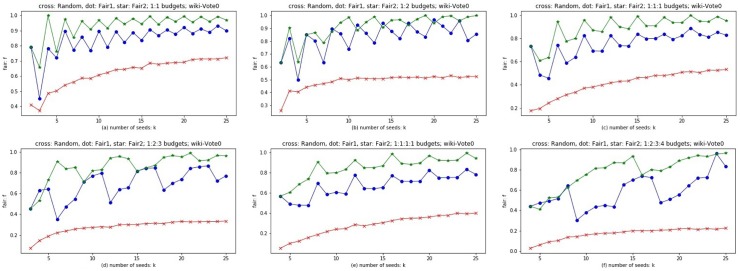
The results of Fair1 and Fair2 on multilayer network Wiki-Vote0. Abscissa is the number of seeds; ordinates represent the value of *f*; red cross, blue dot and green star line represent Random, Fair1 and Fair2 respectively. Subfigure (a)-(f) are of different proportion of budgets.

**Fig 11:**
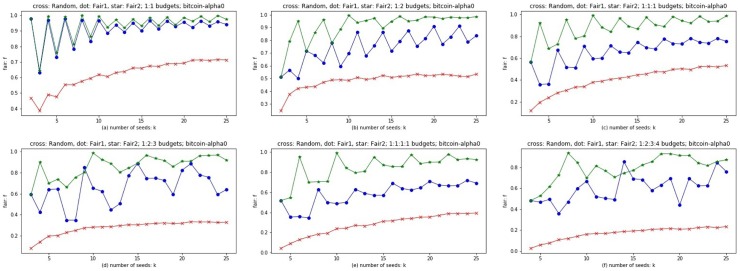
The results of Fair1 and Fair2 on multilayer network Bitcoin-alpha0 abscissa is the number of seeds; ordinates represent the value of *f*; red cross, blue dot and green star line represent Random, Fair1 and Fair2 respectively. Subfigure (a)-(f) are of different proportion of budgets.

From the experimental results shown in Figs 10 and 11, Fair1 and Fair2 significantly outperform random seed allocation, and Fair2 has the best results on different budgets for different networks. According to Theorem 3, both Fair1 and Fair2 are greedy strategies to maximize fairness *f*. The curves of Fair1 and Fair2 are oscillating, but with the increase of the number of seeds *k*, the amplitude of the oscillation decreases. No matter for Fair1 or Fair2, which color the next seed is assigned to is based on the current optimal choice, i.e., allocating the coming seed to color *i* with the minimum *f*_*i*_ (If more than one *f*_*i*_ is equal to the minimum, choose a color *i* from these *f*_*i*_ randomly). If the current *f* is close to 0, i.e., the gap between the minimum and the maximum of {*f*_1_, *f*_2_, …, *f*_*t*_} is very large, then allocating a new coming seed usually increase the value of *f*. On the contrary, if the current *f* is close to 1, allocating a new coming seed usually break the balance among {*f*_1_, *f*_2_, …, *f*_*t*_} and even make *f* smaller. This is the potential reason for the oscillations. With the increase of *k*, i.e., the number of allocated seeds, the ratio of a new coming seed’s influence spread to allocated seeds’ influence spread is usually getting smaller, especially for Fair2, because the influence spread of the newly allocated seed is non-increasing. Therefore, allocating a new coming seed has less effect on *f* with the increase of *k*. This is the potential reason of the amplitude decrement and the convergence of curves with the increase of *k*. The difference between Fair1 and Fair2 is that Fair1 allocates seeds by their influence spread from small to large, while Fair2 allocates seeds by their influence spread from large to small. Recall that, the same allocation strategy of Fair1 and Fair2 is allocating the coming seed to color *i* with the minimum *f*_*i*_ (If more than one *f*_*i*_ is equal to the minimum, choose a color *i* from these *f*_*i*_ randomly). For Fair1, the allocations of the first *t* seeds are all random because the initial values of *f*_*i*_ (*i* = 1, 2, …, *t*) are all equal to zeros. For Fair2, since the initial values of *f*_*i*_ (*i* = 1, 2, …, *t*) are the reciprocals of the budgets respectively, they may be different from each other, i.e., the allocations of the first *t* seeds may not be random. Therefore, Fair2 is more reasonable than Fair1 for the first *t* allocations. On the other hand, as we discussed before, with the increase of *k*, the oscillation of Fair2 decreases faster than that of Fair1. Therefore, Fair2 is superior to Fair1 in almost all cases.

## Conclusion

In this work, we propose a multi-influence diffusion model MMIC for multilayer social networks. Unlike traditional models of single influence in a single network, MMIC model considers not only multilayer networks, but also multiple competing influences propagating within them. From the point of view of the network owner, we propose a Fair Seed Allocation problem for multilayer networks. Firstly, we propose ‘RRE method’ as Algorithm 3 to find the *k* most influential seeds. Then we allocate the *k* seeds to *t* different colors (customers) according to Fair1 (Algorithm 4) or Fair2 (Algorithm 5) so that their influence spread is proportional to their budgets. We proved theoretically that the two allocation strategies are greedy choices. Our experiments on real social networks show the effectiveness of our methods.
